# Polydatin Inhibits Formation of Macrophage-Derived Foam Cells

**DOI:** 10.1155/2015/729017

**Published:** 2015-10-19

**Authors:** Min Wu, Meixia Liu, Gang Guo, Wengao Zhang, Longtao Liu

**Affiliations:** ^1^Department of Cardiovascular Diseases, Guang'anmen Hospital, China Academy of Chinese Medical Sciences, Beijing 100053, China; ^2^Xiyuan Hospital, China Academy of Chinese Medical Sciences, Beijing 100091, China; ^3^Qilu Hospital of Shandong University, Jinan 250012, China; ^4^Shandong University of Traditional Chinese Medicine, Shandong 250355, China

## Abstract

*Rhizoma Polygoni Cuspidati*, a Chinese herbal medicine, has been widely used in traditional Chinese medicine for a long time. Polydatin, one of the major active ingredients in *Rhizoma Polygoni Cuspidati*, has been recently shown to possess extensive cardiovascular pharmacological activities. In present study, we examined the effects of Polydatin on the formation of peritoneal macrophage-derived foam cells in Apolipoprotein E gene knockout mice (ApoE^−/−^) and explored the potential underlying mechanisms. Peritoneal macrophages were collected from ApoE^−/−^ mice and cultured *in vitro*. These cells sequentially were divided into four groups: Control group, Model group, Lovastatin group, and Polydatin group. Our results demonstrated that Polydatin significantly inhibits the formation of foam cells derived from peritoneal macrophages. Further studies indicated that Polydatin regulates the metabolism of intracellular lipid and possesses anti-inflammatory effects, which may be regulated through the PPAR-*γ* signaling pathways.

## 1. Introduction

Complications of atherosclerosis are the leading causes of death throughout the world. Atherosclerosis is a chronic, inflammatory disorder characterized by the deposition of excess lipids in the arterial intima [1–3]. Macrophage-derived foam cells play essential roles in all stages of atherosclerosis [4]. From early fatty-streak lesions to advanced plaques, macrophage-derived foam cells are integral to the development and progression of atherosclerosis. Lipid homeostasis, especially cholesterol homeostasis, plays a crucial role in the formation of foam cells [5]. Macrophage foam cell formation is a prominent feature of human atherosclerotic plaques, usually considered to be correlated to uptake of and inflammatory response to oxidized low density lipoproteins (ox-LDL) [6]. The limited efficacy of current treatment strategies for atherosclerosis and its complications highlights the urgent need for new therapeutic options [7].

Recruitment of macrophages and their subsequent uptake of ox-LDL by scavenger receptors are major cellular events contributing to fatty-streak formation [8]. Among the scavenger receptors, cluster of differentiation antigen 36 (CD36) is known to be the principal receptor in the process of foam cell formation [9, 10]. The most convincing data supporting a critical role of CD36 in foam cell formation and atherosclerosis are from studies of a CD36-null engineered mouse model. Macrophages isolated from CD36-deficient animals are profoundly defective in uptake of ox-LDL and foam cell formation. Accordingly, knockout of CD36 in proatherogenic ApoE-null mice protects the development of atherosclerosis lesions in these animals. Compared to CD36-intact ApoE-null mice fed with a western diet, these animals showed a more than 70% reduction in aortic lesion size [11].

Cholesterol efflux is considered as the most important key point with regard to maintenance of cholesterol homeostasis and atherosclerosis. One of the major potential cholesterol efflux pathways in macrophages is mediated by ATP-binding cassette transporter A1 (ABCA1). It promotes efflux of phospholipids and cholesterol to lipid-poor ApoA-I in a process that involves the direct binding of ApoA-I to the transporter. So ABCA1 was considered as the key mediator of macrophage cholesterol efflux to mature HDL [12]. The intracellular cholesterol homeostasis in macrophages is dynamically regulated by cholesterol uptake and cholesterol efflux, processes that are tightly controlled by these scavenger receptors, such as CD36 and ABCA1 [13].

Peroxisome proliferators-activated receptor gamma (PPAR-*γ*) is a nuclear transcription factor that is highly expressed in macrophages and macrophage-derived foam cells in atherosclerotic lesions. PPAR-*γ* regulates cholesterol metabolism and attenuates inflammation [14]. It inhibits macrophage foam cell formation and atherosclerosis [15]. PPAR-*γ* promotes monocyte/macrophage differentiation and the uptake of ox-LDL by enhancing CD36 expression [16]. PPAR-*γ* is in the first step of the reverse-cholesterol-transport pathway through the activation of ABCA1-mediated cholesterol efflux in human macrophages [17]. Disruption of the PPAR-*γ* gene suppresses the expression of ABCA1 in macrophage and reduces cholesterol efflux. So PPAR-*γ* plays a critical role in the regulation of cholesterol homeostasis by controlling the expression of a group of genes that mediate cholesterol efflux from cells and its transport in plasma [18].

*Rhizoma Polygoni Cuspidati*, a traditional Chinese herbal medicine, was thought to have actions of “dispelling dampness, alleviating jaundice, clearing heat, subsiding toxin, activating blood, and removing stasis” [19]. Polydatin, one of its chief active ingredients, has been shown to possess extensive cardiovascular pharmacological activities in recent pharmacological studies. Polydatin was shown to markedly affect the regulation of blood lipid, protecting cardiomyocytes, dilating blood vessels, antagonizing platelet aggregation, thrombosis, and atherosclerosis [20]. However, the direct effects of Polydatin on the uptake of ox-LDL by macrophages and formation of foam cells have not yet been elucidated. The mechanism of the antiatherosclerotic effects of Polydatin also remains unclear.

To clarify the effect of Polydatin on peritoneal macrophage-derived foam cells of ApoE^−/−^ mice, we investigated the action of Polydatin on the uptake of ox-LDL, the metabolism of intracellular lipid, the expression of inflammatory factors, and mRNA expression of PPAR-*γ*, ABCA1, and CD36 in peritoneal macrophage. Our results demonstrated that Polydatin significantly inhibits the formation of foam cells in peritoneal macrophages. Polydatin has significant anti-inflammatory effects and regulates the metabolism of lipid, possibly through the PPAR-*γ* signaling pathways.

## 2. Materials and Methods

### 2.1. Animals

Six-week-old ApoE^−/−^ mice were purchased from the Jackson Laboratory (USA) and bred by the Laboratory Animal Center of Beijing University, weighing 19 to 21 g.

### 2.2. Reagents and Chemicals

Polydatin was extracted and purified from* Rhizoma Polygoni Cuspidati* by Xi'an Guanyu BioTech Co. Ltd. The purity of Polydatin is 99.42%. Lovastatin was purchased by Beijing Winsunny Co. Ltd. RPMI 1640, fetal bovine serum (FBS), and antibiotics (streptomycin/penicillin) were purchased from Gibco (BRL Life Technologies, Grand Island, NY). TNF-*α* and IL-6 ELISA kits were purchased from Shanghai Westang BioTech Inc., Ltd., Shanghai.

### 2.3. Isolation and Culture of Mouse Peritoneal Macrophages

The ApoE^−/−^ mice were sacrificed by cervical dislocation, in accordance with the Principles of Laboratory Animal Care (NIH publication number 85-23, revised 1985) and the Guidelines of the Animal Investigation Committee of Peking University. Sterile ice-cold phosphate-buffered saline (PBS) was injected into the peritoneal cavity of each mouse and peritoneal lavage was performed. This fluid was carefully collected and centrifuged at 1,000 rpm for 6 min. After centrifugation, the supernatant was then discarded, and the cell pellet was resuspended in RPMI 1640 medium (containing 100 IU/mL of penicillin, 100 *μ*g/mL of streptomycin, and 100 *μ*g/mL of l-glutamine) and plated in 6-well tissue culture plates (Costar) at 1.5 × 10^6^ cells per well. Cells were incubated in a humidified atmosphere of 5% CO_2_ at 37°C for 2-3 h to allow adherence, and nonadherent cells were rinsed away with prewarmed RPMI 1640 medium and 2 mL of complete RPMI 1640 medium (supplemented with 10% fetal bovine serum) was added. Media with all additions were replaced daily and macrophages were used within 5 days from harvesting for further analysis [21, 22].

### 2.4. LDL Modification

LDL was exposed to 5 *μ*mol/L CuSO_4_ for 24 hours at 37°C. Cu^2+^ was then removed by extensive dialysis. The extent of modification was determined by measurement of thiobarbituric acid-reactive substances. ox-LDL containing 30–60 nmol thiobarbituric acid-reactive substances defined as malondialdehyde equivalents per milligram of LDL protein was used for experiments [23].

### 2.5. Groups

Peritoneal macrophages of ApoE^−/−^ mice were collected and divided into 4 groups: Control group (treated with calf serum 250 *μ*L), Model group (treated with calf serum 250 *μ*L and ox-LDL 250 *μ*g), Lovastatin group (treated with calf serum 250 *μ*L, ox-LDL 250 *μ*g, and 110 *μ*g/mL Lovastatin), and Polydatin group (treated with calf serum 250 *μ*L, ox-LDL 250 *μ*g, and 8.9 *μ*g/mL Polydatin). Culture fluid was added to make up for 2.5 mL in all groups.

### 2.6. Ultrastructural Structure Observation of Macrophage Cells

After being loaded with ox-LDL, Polydatin, and Lovastatin for 48 h, those macrophages were centrifuged at 400 g for 10 min, and the pellets produced were washed with PBS and then mixed with 2% (v/v) P-formaldehyde-glutaraldehyde in 0.05 M cacodylate buffer (pH 7.4) for 4 hours at 4°C. After that, the pellets were prepared for transmission electron microscope (JEM-1200EX).

### 2.7. Intracellular Lipid Analysis in Macrophages

Intracellular lipids in macrophages were extracted with hexane/isopropyl alcohol (3 : 2) after 0, 24, and 48 h of incubation with ox-LDL, Polydatin, and Lovastatin, evaporated, and then dissolved in isopropyl alcohol containing 10% Triton X-100 for preparation of a sample solution. Free cholesterol and total cholesterol were determined by commercial assay systems (Wako Chemicals). Cholesterol ester was estimated by subtracting free cholesterol from total cholesterol. The experiment was repeated at three times.

### 2.8. Protein Levels of TNF-*α* and IL-1*β* Detection with ELISA

After 24 h and 48 h of incubation with ox-LDL, the levels of TNF-*α* and IL-6 in the cultured media were measured by ELISA kits according to the manufacturer's instructions (Shanghai Westang BioTech Inc., Ltd., Shanghai). The ELISA data were obtained at least at three independent experiments.

### 2.9. RT-PCR Analysis of Expression of PPAR-*γ*, ABCA1, and CD36 mRNA

The total RNA was extracted from cultured peritoneal macrophages using RNA isolation kit (Sangong BioTech Co., Ltd., Shanghai), according to the manufacturer's instructions. cDNAs were prepared from 0.3 *μ*g of RNA.

RT-PCR was performed according to the manufacturer's instructions, using oligonucleotide primers to detect PPAR-*γ*, ABCA1, and CD36 mRNA (Sangong BioTech Co., Ltd., Shanghai), PPAR-*γ* (403 bp): sense 5′-CCC TGG CAA AGC ATT TGT AT-3′, antisense 5′-AAT CCT TGG CCC TCT GAG AT-3′; ABCA1 (364 bp): sense 5′-CAG ATG CCC TAC CCC TGT TA-3′, antisense 5′-GGG AGA AGA GCG TGC TAA TG-3′; CD36 (418 bp): sense 5′-CCT TAA AGG AAT CCC CGT GT-3′, antisense 5′-CCA ATG GTC CCA GTC TCA TT-3′; *β*-actin (302 bp): sense 5′-TCC TCC CTG GAG AAG AGC TA-3′, antisense, 5′-TCA GGA GGA GCA ATG ATC TTG-3′ [24]. This experiment was repeated at least three times.

### 2.10. Statistical Analysis

The results are expressed as mean ± SD values for the number of experiments. Statistical significance was compared in each treated group with the negative control and determined by one-way ANOVA test. Each experiment was repeated at least three times. SPSS version 13.0 (SPSS Inc., IL, USA) was used for analysis. Values with *P* < 0.05 were considered significant.

## 3. Results

### 3.1. Polydatin Protects the Ultrastructure of Peritoneal Macrophage Cells

**Figure 1:**
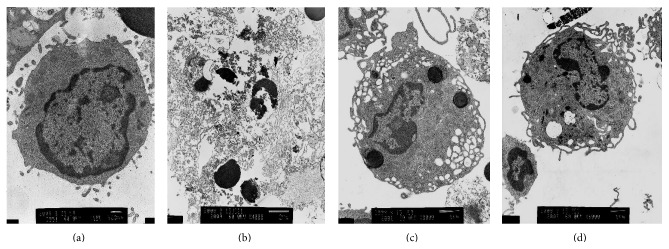
Polydatin protects the ultrastructure of peritoneal macrophage. (a) Control group, (b) Model group, (c) Lovastatin group, (d) Polydatin group.

To examine the potential effects of Polydatin on macrophage cells, the mouse macrophages were incubated with ox-LDL for 48 h in the absence or presence of Polydatin and the ultrastructure was evaluated under electron microscope. The normal structure of macrophage is shown in Figure 1(a). In comparison, the structure of macrophages in the Model group was completely damaged and the organelles in the periplasm disappeared. The nucleus split into multiple shivers with different sizes. The structure of the nuclear membrane, nucleoli, and euchromatin disappeared (Figure 1(b)). In Polydatin and Lovastatin group, the structure of nucleus was integrated. The nuclear membrane, nucleolus, and abundant euchromatin were observed. A part of the organelles was damaged and vacuolization was observed. These suggested that peritoneal macrophage of ApoE^−/−^ mice had favorable response and phagocytosis ability to ox-LDL. Combined treatment with Polydatin/Lovastatin and ox-LDL significantly reduced the damage of ox-LDL on intracellular ultrastructure in macrophages compared to the group of ox-LDL treatment alone (Figures 1(c) and 1(d)).

### 3.2. Polydatin Regulates the Metabolism of Intracellular Lipid in Peritoneal Macrophage Cells

**Figure 2:**
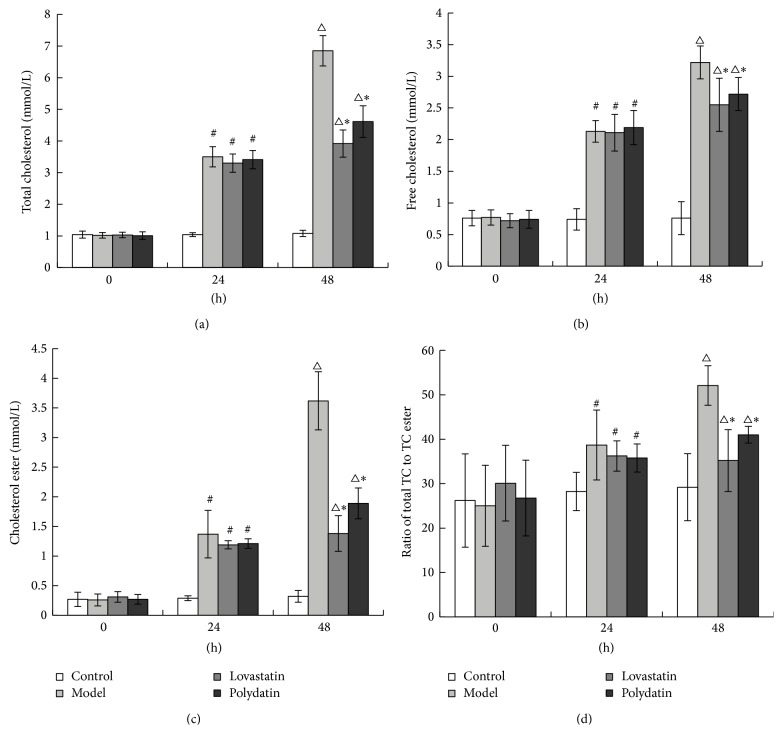
Polydatin regulates the metabolism of intracellular lipid in peritoneal macrophage cells. (a) Level of total cholesterol, (b) level of free cholesterol, (c) level of cholesterol ester, (d) ratio of cholesterol ester to total cholesterol. ^#^ *P* < 0.01, ^△^ *P* < 0.01, compared with the Control group. ^*∗*^ *P* < 0.01, compared with the Model group.

After being loaded with ox-LDL for 24 h and 48 h, the levels of total cholesterol, free cholesterol, and cholesterol ester in macrophages in Model group were elevated. The ratio of cholesterol ester to total cholesterol in macrophage was more than 50% in Model group, which is in accordance with the pathological change of foam cells. After treatment for 24 h and 48 h, Polydatin and Lovastatin significantly reduced cholesterol accumulation in ox-LDL loaded macrophages. There was no difference observed between Polydatin and Lovastatin group (Figures 2(a)–2(d)).

### 3.3. Polydatin Reduces Protein Levels of TNF-*α* and IL-1*β* in Peritoneal Macrophage Cells

**Figure 3:**
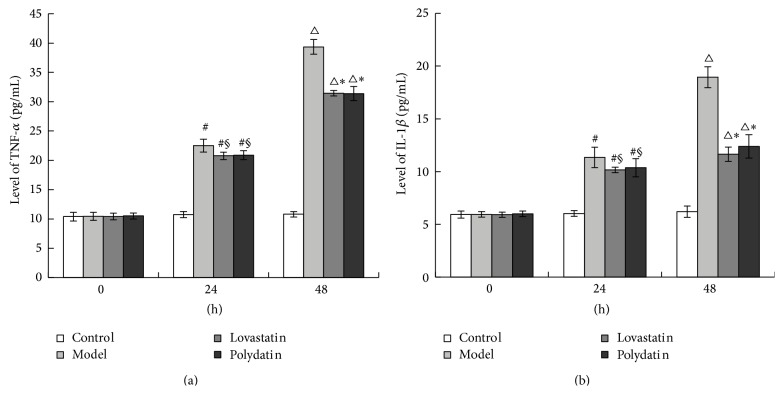
Polydatin reduces the protein level of TNF-*α* and IL-1*β* in peritoneal macrophage cells. (a) TNF-*α* level; (b) IL-1*β* level. ^#^ *P* < 0.01, ^△^ *P* < 0.01, compared with the Control group. ^§^ *P* < 0.05, ^*∗*^ *P* < 0.01, compared with the Model group.

To further investigate the potential effect of Polydatin on inflammatory factors, the expression of TNF-*α* and IL-1*β* in the peritoneal macrophage cells was evaluated by ELISA. After ox-LDL treatment for 24 h and 48 h, the protein level of TNF-*α* and IL-1*β* was significantly increased. Polydatin and Lovastatin reduced the expression of the TNF-*α* and IL-1*β* levels. And there was no difference between Polydatin and Lovastatin group (Figures 3(a) and 3(b)). This illustrated that chemotropism of peritoneal macrophages in ApoE^−/−^ mice is enhanced by ox-LDL, accompanied by produce of abundant inflammatory factors, such as TNF-*α* and IL-1*β*.

### 3.4. Polydatin Regulates mRNA Expression of PPAR-*γ*, ABCA1, and CD36 in Peritoneal Macrophage Cells

**Figure 4:**
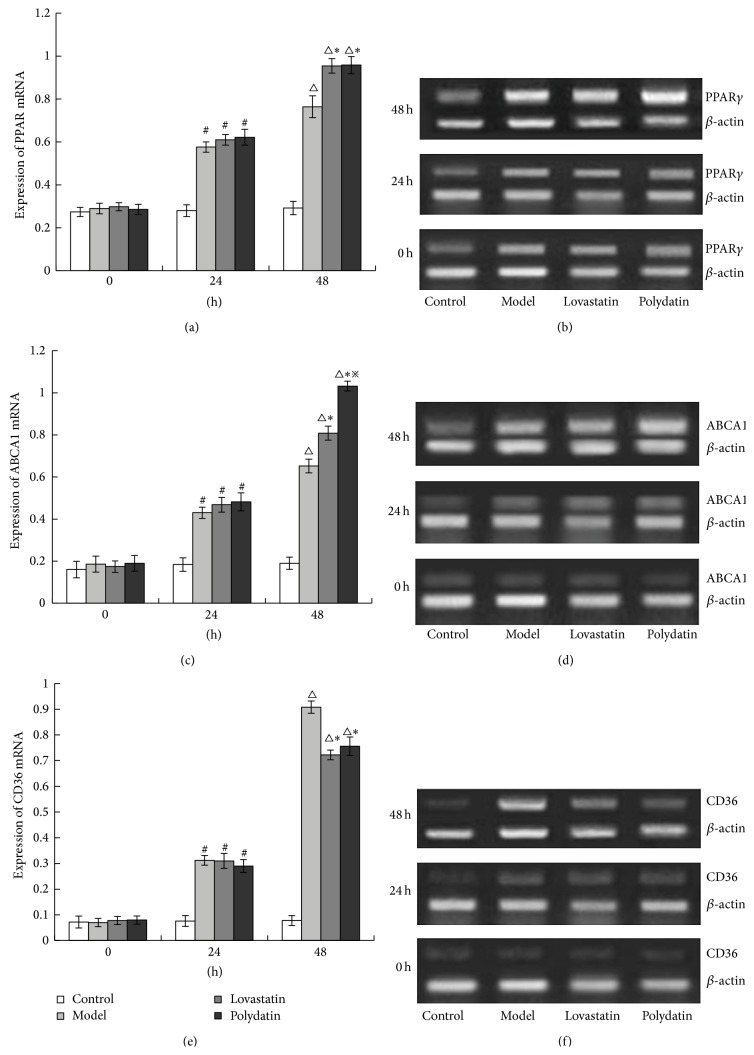
Polydatin regulates mRNA expression of PPAR-*γ*, ABCA1, and CD36 in peritoneal macrophage cells. Figures (b), (d), and (f) are the mRNA expression of PPAR-*γ*, ABCA1, and CD36. (a), (c), and (e) are quantitated from these results. ^#^ *P* < 0.01, ^△^ *P* < 0.01, compared with the Control group. ^*∗*^ *P* < 0.01, compared with the Model group, ^*※*^ *P* < 0.05, compared with the Lovastatin group.

The mRNA expression of PPAR-*γ*, ABCA1, and CD36 was examined by RT-PCR to determine whether Polydatin has a suppressive effect on the PPAR-*γ* pathway in the peritoneal macrophage cells. After ox-LDL treatment for 24 h and 48 h, the mRNA levels of PPAR-*γ*, ABCA1, and CD36 were remarkably increased in peritoneal macrophage cells. However, the mRNA expression of PPAR-*γ* and ABCA1 was found to increase in Polydatin and Lovastatin group after 24 and 48 h treatment, compared to the ox-LDL treatment group. But the mRNA expression of CD36 was reduced significantly in Polydatin and Lovastatin group after 24 h treatment. There was no difference in the mRNA expression of PPAR-*γ* and CD36 between the Polydatin and Lovastatin group. But the mRNA expression of ABCA1 in Polydatin group was higher than that in Lovastatin group (*P* < 0.05) (Figures 4(a)–4(f)).

## 4. Discussion

Atherosclerosis begins early in life and frequently leads to severe complications in later life with high morbidity and mortality. Macrophage cholesterol accumulation-induced foam cell formation is the hallmark of early atherosclerosis [25]. Elucidation of molecular and cellular processes involving macrophages has led to numerous therapeutic targets being suggested [7]. Cholesterol accumulation in macrophages can result from an unbalanced cellular cholesterol flux, increased uptake of atherogenic lipoproteins, and/or decreased cholesterol efflux from the cells [26–28]. In our study, we showed that Polydatin could increase cholesterol efflux from macrophage and decrease the uptake of ox-LDL, thus resulting in the inhibition of cholesterol accumulation in macrophage. Its mechanism is possibly induced in a PPAR-*γ*-dependent manner.

Large sample clinical trials have confirmed that the statins have antiatherosclerotic effects, including improving endothelial function, suppressing platelet aggregation, increasing anti-inflammatory effects, and stabilizing atherosclerotic plaque [29–32]. However, the statins result in some significant side effects. So we try to find the new medicine from Chinese herbs which have the antiatherosclerotic effects and regulate lipid, but with few side effects. The search for new drugs with antiatherosclerosis and lipid-regulation effects has gained momentum over the years, resulting in numerous reports on significant activities of natural agents. Many classes of dietary components and natural compounds have been tested to regulate serum lipid concentrations with the aim of lowering the incidence of atherosclerosis and coronary heart disease [20, 33]. Our research group found that Polydatin can decrease carotid intima-media thickness (IMT), plaque integral and reduce the level of plaque stability related serum indexes such as Hs-CRP, MMP-1, and TIMP in patients with carotid atherosclerosis [34, 35].

*Rhizoma Polygoni Cuspidati* is recorded first in the book named* Miscellaneous Records of Famous Physicians* (Ming Yi Bie Lu). Modern pharmacological studies have shown that it has obvious antibacterial, anti-inflammation, diuresis, purgation, and menstrual restoration effects [36]. As one of its main ingredients, Polydatin has multiple biological actions, such as liver protection, anti-inflammation, antitumor, and antipathogenic microbe, and is applied to prevent/treat cardiovascular diseases. Particularly, its cardiovascular pharmacological actions, such as cardiomyocyte (CM) protection, vascular smooth muscle dilation, platelet aggregation, thrombosis, and atherosclerosis prevention, have received great attention from scholars of related fields in latest years [37].

Cholesterol-loaded macrophages or foam cells are a major contributor to the atherosclerotic plaque.* In vitro* studies have shown that modified forms of low density lipoprotein cause accumulation of cholesterol esters in cultured macrophages [38]. In this study, we collected the peritoneal macrophages from ApoE^−/−^ mice, which have become the most widely used rodent model for the study of atherosclerosis [39, 40]. Then we stimulated the macrophage cells by ox-LDL to get the “foam cell” model. The results showed that the ratio of cholesterol ester to total cholesterol in macrophage is more than 50% in Model group and this is consistent with the pathological change of foam cells. We used the MTT analysis method to decide the appropriate dosage of Polydatin and Lovastatin (data not showed). In our study, we found that combined treatment with Polydatin/Lovastatin and ox-LDL significantly relieve the damage of ox-LDL on intracellular ultrastructure in macrophages compared with the ox-LDL loaded cells. Polydatin and Lovastatin regulate the metabolism of intracellular lipid and so confer the protective role in the formation of macrophage foam cells.

In the study, we found that, after incubation with ox-LDL for 24 h and 48 h, peritoneal macrophage of ApoE^−/−^ mice had favorable responsibility and phagocytosis ability to ox-LDL and abundant inflammatory factors were secreted. Working together, these effects could inhibit the elevation of TNF-*α*, IL-1*β*. We did not observe any difference between the Polydatin and Lovastatin group, suggesting the efficacy of Polydatin is comparable to Lovastatin.

CD36 and ABCA1 are the downstream target genes which are transcriptionally controlled by PPAR-*γ*. CD36 is correlated to intracellular cholesterol accumulation. ABCA1 can promote diffluence of free cholesterol and phosphatides from cells. The activated PPAR-*γ* by ox-LDL can induce CD36 expression in the macrophage, promote the uptake of ox-LDL, increase intracellular cholesterol accumulation, and then motivate foam cell formation [16]. This is the positive feedback pathway. At the same time, the activated PPAR-*γ* also upregulates ABCA1 expression, reduces cholesterol, and then inhibits foam cell formation. This is the negative feedback pathway. Therefore, the regulation of the ox-LDL-PPAR-*γ*-CD36 and ox-LDL-PPAR-*γ*-ABCA1 signaling pathways is important for inhibition of foam cell formation and antiatherosclerosis [41]. In our study, we examined whether the expression of PPAR-*γ* affects the expression of ABCA1 and CD36. After treating with ox-LDL, Polydatin, and Lovastatin for 24 h and 48 h, the expression of PPAR-*γ*, ABCA1, and CD36 at mRNA levels was increased. Polydatin and Lovastatin might upregulate the mRNA expression of PPAR-*γ* and ABCA1 and downregulate the mRNA expression of CD36. This suggests that Polydatin might regulate the expression of ABCA1 mRNA and CD36 mRNA through activating the PPAR-*γ* signaling, which sequentially inhibit the formation of macrophage foam cells.

In conclusion, our studies have indicated that Polydatin inhibits the formation of peritoneal macrophage-derived foam cells of ApoE^−/−^ mice. Further investigation has shown that Polydatin has anti-inflammatory effects and regulates the metabolism of intracellular lipid, possibly through the PPAR-*γ* signaling pathway. Thus, our data have provided some experimental evidences to use Polydatin in prevention and cure of atherosclerosis.
